# Evaluation of decontamination methods of oral biofilms formed on screw-shaped, rough and machined surface implants: an ex vivo study

**DOI:** 10.1186/s40729-020-00212-y

**Published:** 2020-04-22

**Authors:** Motohiro Otsuki, Masahiro Wada, Masaya Yamaguchi, Shigetada Kawabata, Yoshinobu Maeda, Kazunori Ikebe

**Affiliations:** 1 Department of Prosthodontics, Gerodontology and Oral Rehabilitation, Osaka University Graduate School of Dentistry, 1-8 Yamadaoka, Suita, Osaka, 565-0871 Japan; 2 Department of Oral and Molecular Microbiology, Osaka University Graduate School of Dentistry, Osaka, Japan

**Keywords:** Dental implant, Decontamination, Biofilms, Rough surface implant, Machined surface implant, Peri-implantitis, Screw-shaped implant

## Abstract

**Background:**

To evaluate the effect of several representative decontamination methods of oral biofilms on different implant surfaces.

**Material and methods:**

Eleven participants wore a hard resin splint carrying 6 rough (GC Aadva**®** implant; 3.3-mm diameter, 8-mm length) or machined (not commercially available) surface implants for 4 days to accumulate dental plaque naturally on the titanium surfaces of the implants. Apart from surface roughness, the morphology of all implants was identical. After detaching the implants from the splints, the ability of the following decontamination methods—gauze soaked in saline (G), ultrasonic scaler (US), air abrasive (Air), rotary stainless steel instrument (Rot), and Er:YAG laser (Las)—to cleanse the contaminated implant surface for 1 min extra-orally was tested. The control (Cont) group did not receive any decontamination. Scanning electron microscopic (SEM) investigation of one participant’s samples was employed to examine the post-instrumented implant surface for qualitative analysis, and bacterial culture of the remaining 10 participants’ samples was performed to count the number of colony-forming units (CFU) for quantitative analysis. The experimental sequence was initially performed for the rough surface implants and then similarly repeated for the machined surface implants. Bacterial CFU counts among the six groups were analyzed using the Steel-Dwass test, and differences between rough and machined surface implants were determined using the Mann-Whitney *U* test.

**Results:**

G and Rot eliminated most biofilms on machined surface implants according to SEM analysis. G, Air, and Rot removed significantly more of the biofilms on rough and machined surface implants compared with US according to CFU counts. Moreover, G significantly reduced more biofilms than Las on machined surface implants. The analysis between rough and machined surface implants showed that Cont, G, and US were better able to cleanse biofilms on machined surface implants compared with rough surface implants.

**Conclusions:**

Gauze soaked in saline and rotary stainless steel instruments may be advantageous for cleansing contaminated implant surfaces based on the qualitative and quantitative analyses. In contrast, air abrasives were not shown to be preferable in the qualitative analyses. Additionally, apart from the Er:YAG laser, the reduction of biofilms assessed in both qualitative and quantitative analyses demonstrated that all decontamination methods were better at cleansing machined surface implants compared with rough surface implants.

## Background

Dental implants are now used broadly for recovering loss of masticatory function and esthetics. Although it has been revealed that dental implants can often survive long term, biological and mechanical complications may arise. Recent cross-sectional and retrospective studies noted a higher prevalence of peri-implantitis at the implant and subject level than previously considered [1–4]. Additionally, once peri-implantitis occurred, it was more destructive and progressed rapidly compared with periodontitis because of different histological features (e.g., lack of healthy connective tissue seen around the teeth separating inflammatory cells from bone) [5]. Moreover, the predictability of peri-implantitis treatment is not as high as that of periodontitis treatment [6, 7]. The success of peri-implantitis treatment is associated with multiple factors, such as implant surface [6], disease severity [7], smoking habit [8], and surgeon’s experience [9]. However conflicting results were observed in experimental studies focused on biocompatibility after cleansing contaminated titanium disks or implants [10–13]. To date, a standard technique for cleansing contaminated surfaces affected by peri-implantitis has not yet been established clinically or even experimentally. Additionally, the titanium disk or other forms of titanium used in most in vitro studies to evaluate decontamination methods do not resemble the screw-shaped implants typically placed in clinical practice in terms of macro- and microstructures, rendering it difficult to extrapolate such results to the clinical setting. Moreover, a single bacterial species or artificial biofilms were often used to evaluate the efficacy of decontamination methods on the materials [14, 15]. The aim of this study is to evaluate the effect of several representative decontamination methods on biofilms formed intraorally on the implant surface using SEM for qualitative analysis and culture technique for quantitative analysis.

## Materials and methods

### Study subjects

Eleven participants, nine men and two women between 28 and 42 years of age (mean age 31.3 ± 4.6), were recruited as the study subjects. All participants provided informed consent verbally. Study information was disseminated to participants both verbally and in written form. The inclusion criteria were as follows:

- Generally healthy subjects
- Dentate subjects without ill-fitting restorations (≥ 24 teeth including both first upper molars)
- No dental treatment planned during the study
- Non-smoker or no use of tobacco for at least 6 months prior to the study enrolment
- No use of systemic antibiotics in the 6 months prior to the study enrolment or daily use of mouthwashes

### Study design

**Fig. 1:**
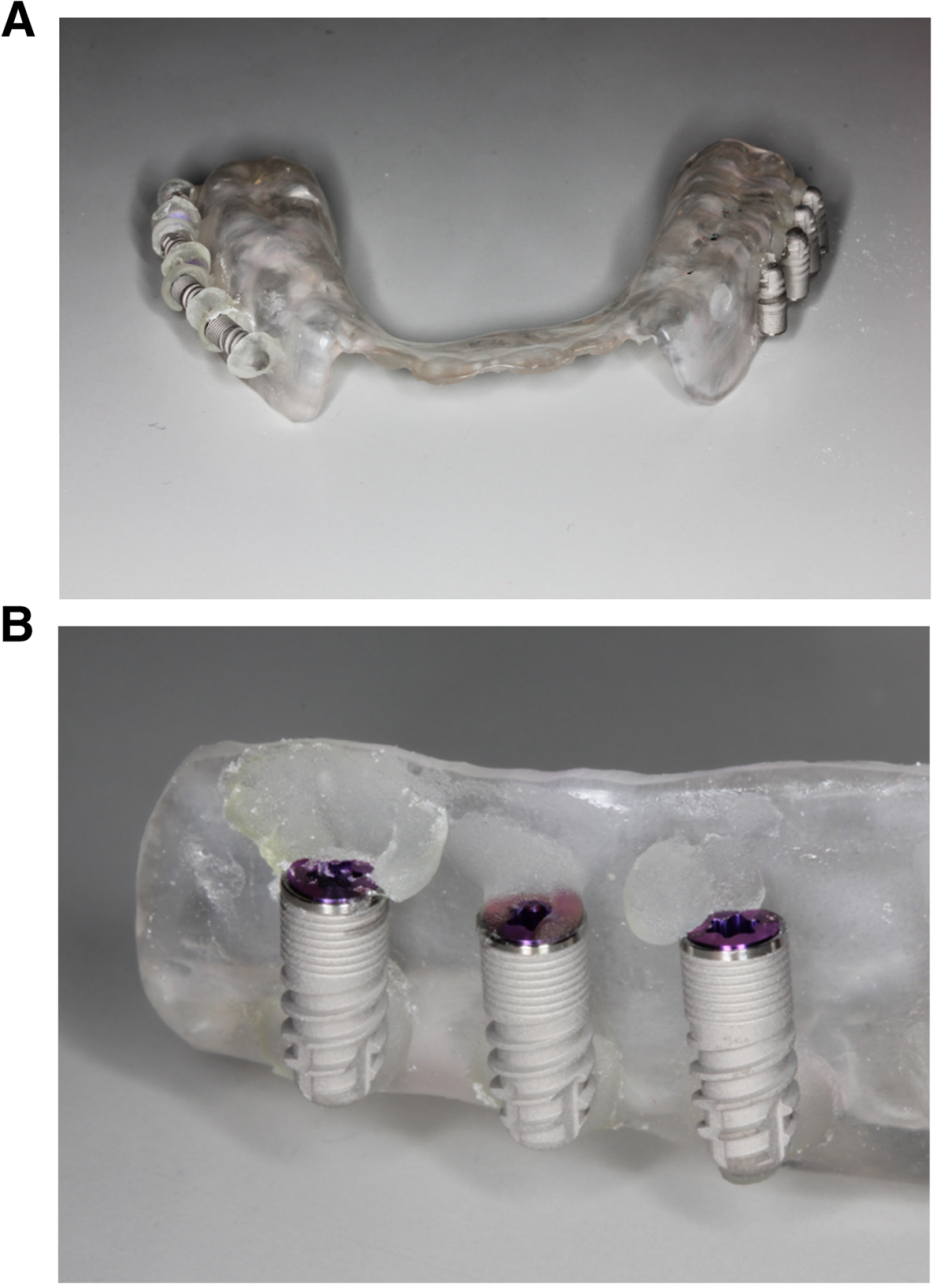
Hard resin splint model carrying 6 implants

**Fig. 2:**
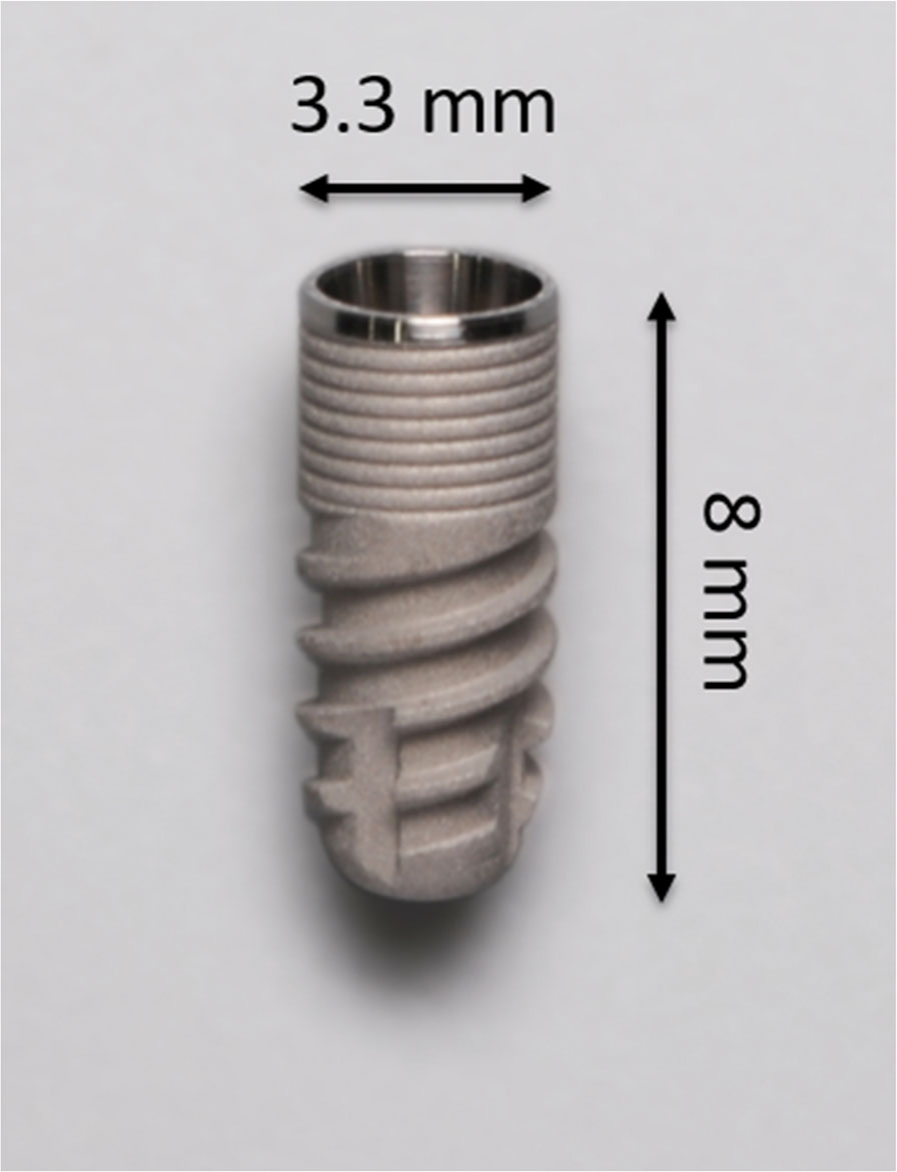
GC Aadva**®** implant; 3.3-mm diameter, 8-mm length

**Fig. 3:**
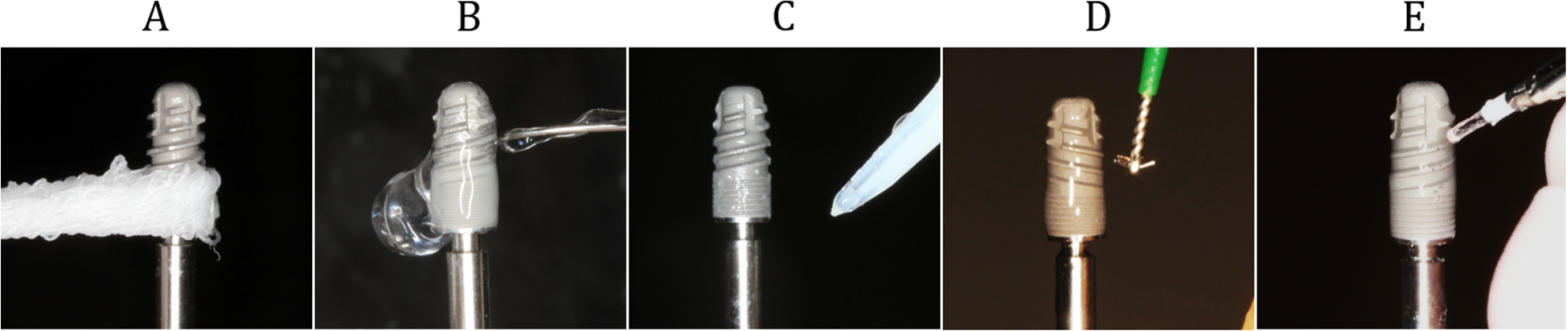
Decontamination methods. **a** Gauze soaked in saline applied using a sawing motion. **b** Ultrasonic scaler (SUPRASSON P-MAX, Satelec-Acteon group, Bordeaux, France, power setting: P5, tip: Implant Protect IP3L/R). **c** Air abrasives (AIR-FLOW MASTER PIEZON**®**, EMS, Nyon, Switzerland, power setting: water flow 100%, air pressure 75%, powder: AIR-FLOW**®** PERIO POWDER, nozzle: PERIO-FLOW**®** nozzles, distance from the nozzle to the implant 2 mm). **d** Rotary stainless steel instrument (iBrush, NeoBiotech©, Los Angeles, USA, rotating speed 1500 rpm). **e** Er:YAG laser (Erwin AdvErL, J.Morita©, Kyoto, Japan, power setting 60 mJ/pulse, 10 pps, tip: C600F, distance from the tip to the implant 2 mm)

An impression of the upper jaw from each participant was taken to create a hard resin splint (Palapress® vario, Heraeus Kulzer, Wehrheim, Germany), and six implants were mounted on both buccal aspects of each splint and bonded with resin caps that could hold the top and apex of the implant (Fig. 1). The implants used in this study were commercially available rough surface implants (GC Aadva® implant, GC, Tokyo, Japan, 3.3-mm diameter, 8-mm length, Sa value 2.0–2.3) and machined surface implants that were identical in design other than the surface roughness (not commercially available, Sa value 0.3–0.5) (Fig. 2). When the implants were mounted on the splint, the cover screws were tightened to prevent plaque accumulation inside the implant bodies. The participants were instructed to wear the splints for 24 h/day during the 4-day experimental period except for mealtimes. During eating and drinking, the splint was taken off and kept in a provided splint box to avoid drying. The participants were allowed to brush their teeth twice a day but were not allowed to use any kind of mouthwash during the entire experimental period. At the end of the 4-day experimental period, the implants were carefully removed from the splints by breaking the resin caps that held them in place. Each implant was randomly assigned to a treatment method (Cont, control (no decontamination); G, gauze soaked in saline; US, ultrasonic scaler (SUPRASSON P-MAX, Satelec-Acteon Group, Bordeaux, France; power setting: P5, tip: Implant Protect IP3L/R); Air, air abrasive (AIR-FLOW MASTER PIEZON®, EMS, Nyon, Switzerland; power setting: water flow 100%, air pressure 75%, powder: AIR-FLOW® PERIO POWDER, nozzle: PERIO-FLOW® nozzles, distance from the nozzle to the implant 2 mm); Rot, Rotary stainless steel instrument (iBrush, NeoBiotech©, Los Angeles, CA, USA; rotating speed 1500 rpm); Las, Er:YAG laser (Erwin AdvErL, J.Morita©, Kyoto, Japan; power setting 60 mJ/pulse, 10 pps, tip C600F, distance from the tip to the implant 2 mm) according to the random number table generated by a spreadsheet software (random number table generator: Excel® for Mac 2011, version 14.7.2, Microsoft®) (Fig. 3). All methods, including Cont, were applied to each set of 6 implants. One investigator (M.O) was blinded to which implant was assigned to which method. The investigator was experienced in using each method to treat peri-implantitis in clinical practice. The implant driver was connected to the implants to hold them without touching the implant surface during decontamination. The implants were decontaminated by their assigned method, apart from those assigned to the Cont group, for 1 min. After cleansing, decontaminated implants were immediately stored in a phosphate-buffered saline solution. Qualitative SEM analysis was performed using samples taken from the participant who had shown average plaque accumulation on the implant surface in the preliminary study (data not shown), and quantitative colony-forming unit (CFU) counts were performed using the remaining 10 participants’ samples. This study protocol was approved by the ethical committee of Osaka University (H26.E-36).

### SEM analysis

The SEM analysis was performed as previously described [16–18]. The decontaminated implant samples were fixed with 2% glutaraldehyde-RPMI 1640 immediately for 1 h at room temperature and washed with distilled water. Then, the samples were dehydrated with 100% *t*-butyl alcohol and freeze-dried. Finally, the samples were coated with platinum and examined using an emission-scanning electron microscope (JSM-6390LVZ; JEOL Ltd., Tokyo, Japan). An ordinal scale with the following variables was set to facilitate the evaluation of cleansability qualitatively by each method.

- No effect: Surface was cleansed ineffectively and covered with an enormous amount of amorphous material, debris, and bacteria.
- Fair: Surface was cleansed partially well but was far from the ideal and covered with a certain amount of amorphous material, debris, and bacteria.
- Good: Surface was cleansed effectively but not perfectly and covered with a little amount or partially no amount of amorphous material, debris, and bacteria.
- Excellent: Surface was cleansed ideally and covered with no or only a small amount of amorphous material, debris, and bacteria.

SEM images were evaluated by two examiners (M.Y. and S.K.) who were unaware of the aim of this study. Kendall’s coefficient of concordance was used to investigate the inter-examiner reliability in the evaluation of SEM images. There was no significant difference between the two examiners (*w* = 0.865, *p* < 0.01).

### Bacterial CFU counts

To examine bacterial amounts on the implant surfaces, the samples were vortexed at maximum power for 30 s in 1 ml phosphate-buffered saline solution. Resuspended bacteria were serially diluted in a phosphate-buffered saline solution and plated on Brain Heart Infusion agar (Becton Dickinson, Sparks, MD, USA). The number of CFUs was counted after overnight growth on the BHI agar at 37 °C in a candle jar.

### Statistical analysis

Statistical significance of differences in bacterial CFU counts among the six groups, including the control group, was analyzed using the Steel-Dwass test (R version 3.4.0 (R Foundation for statistical Computing, Vienna, Austria)). Significant differences between rough and machined surface implants were analyzed using the Mann-Whitney *U* test (SPSS 23.0 (SPSS Inc., Chicago, IL)). *p* < 0.05 was considered statistically significant.

## Results

### Complications

During the experiment, three participants experienced small ulcers caused by the implants carried on the splints; however, it did not affect their daily life. Additionally, there were no signs of gingival inflammation in any participant.

### SEM analysis (Fig 4, Tables 1 and 2)

#### Rough surface implants

G and Rot achieved relatively clean implant surfaces compared with Las in micro- and macrothread areas. US and Air demonstrated fair cleansability in microthread and good cleansability in macrothread areas, whereas Las did not show effective cleansability in either areas.

**Fig. 4:**
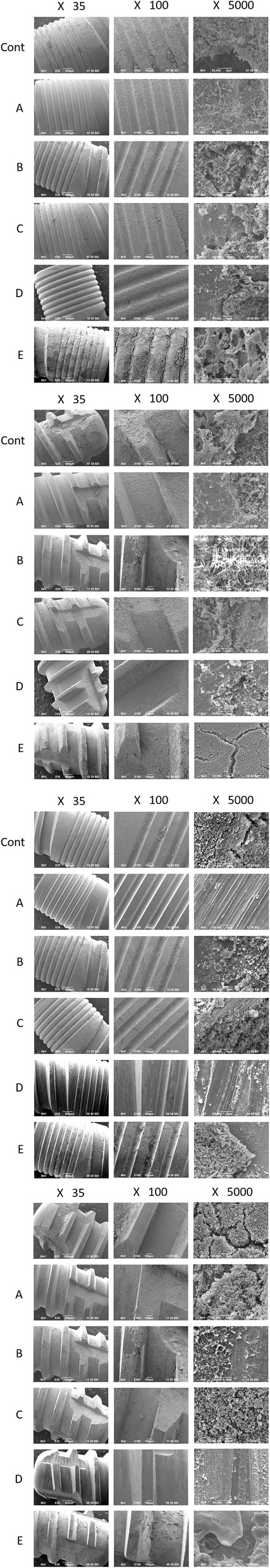
SEM analysis of 4 areas. 1 Rough surface—microthread area. 2 Rough surface—macrothread area. 3 Machined surface—microthread area. 4 Machined surface—macrothread area

**Table 1:** Qualitative evaluation by SEM analysis of micro- and macrothread areas of rough surface implants

**Rough surface (microthread)**	**No effect**	**Fair**	**Good**	**Excellent**
**G**			+	
**US**		+		
**Air**		+		
**Rot**			+	
**Las**	+			
**Rough surface (macrothread)**	**No effect**	**Fair**	**Good**	**Excellent**
**G**			+	
**US**			+	
**Air**			+	
**Rot**			+	
**Las**	+			

No effect: Surface was cleansed ineffectively and covered with an enormous amount of amorphous material, debris and bacteria

Fair: Surface was cleansed partially well but far from the ideal and covered with certain amount of amorphous material, debris, and bacteria

Good: Surface was cleansed effectively but not perfect and covered with a little amount or partially no amount of amorphous material, debris, and bacteria

Excellent: Surface was cleansed ideally and covered with no or only a small amount of amorphous materials, debris, and bacteria

**Table 2:** Qualitative evaluation by SEM analysis of micro- and macrothread areas of machined surface implants

**Machined surface (microthread)**	**No effect**	**Fair**	**Good**	**Excellent**
**G**				+
**US**			+	
**Air**			+	
**Rot**				+
**Las**		+		
**Machined surface (macrothread)**	**No effect**	**Fair**	**Good**	**Excellent**
**G**				+
**US**			+	
**Air**			+	
**Rot**				+
**Las**			+	

No effect: Surface was cleansed ineffectively and covered with an enormous amount of amorphous material, debris and bacteria

Fair: Surface was cleansed partially well but far from the ideal and covered with certain amount of amorphous material, debris, and bacteria

Good: Surface was cleansed effectively but not perfect and covered with a little amount or partially no amount of amorphous material, debris, and bacteria

Excellent: Surface was cleansed ideally and covered with no or only a small amount of amorphous materials, debris, and bacteria

#### Machined surface implants

G and Rot attained almost clean implant surfaces compared with the other methods in micro- and macrothread areas. US and Air also showed good cleansability in micro- and macrothread areas. Las demonstrated fair to good cleansability in both areas.

#### Rough vs machined surface implants

Generally, biofilms appeared to be denser and more firmly attached to rough surface implants than machined surface implants before and after decontamination. Moreover, after cleansing, the machined surface implants appeared cleaner with thin layers and clusters of residual biofilms compared with rough surface implants. Overall, all methods tended to show better cleansability of machined surface implants than rough surface implants.

### Analysis of bacterial CFU count (Figs. 5 and 6, Table 3)

#### Rough surface implants (Fig. 5, Table 3)

All decontamination methods showed significant residual bacterial reduction in terms of bacterial CFU count compared with Cont (*p* < 0.05). Moreover, G, Air, and Rot displayed significantly superior cleansability to US (*p* < 0.05).

**Fig. 5:**
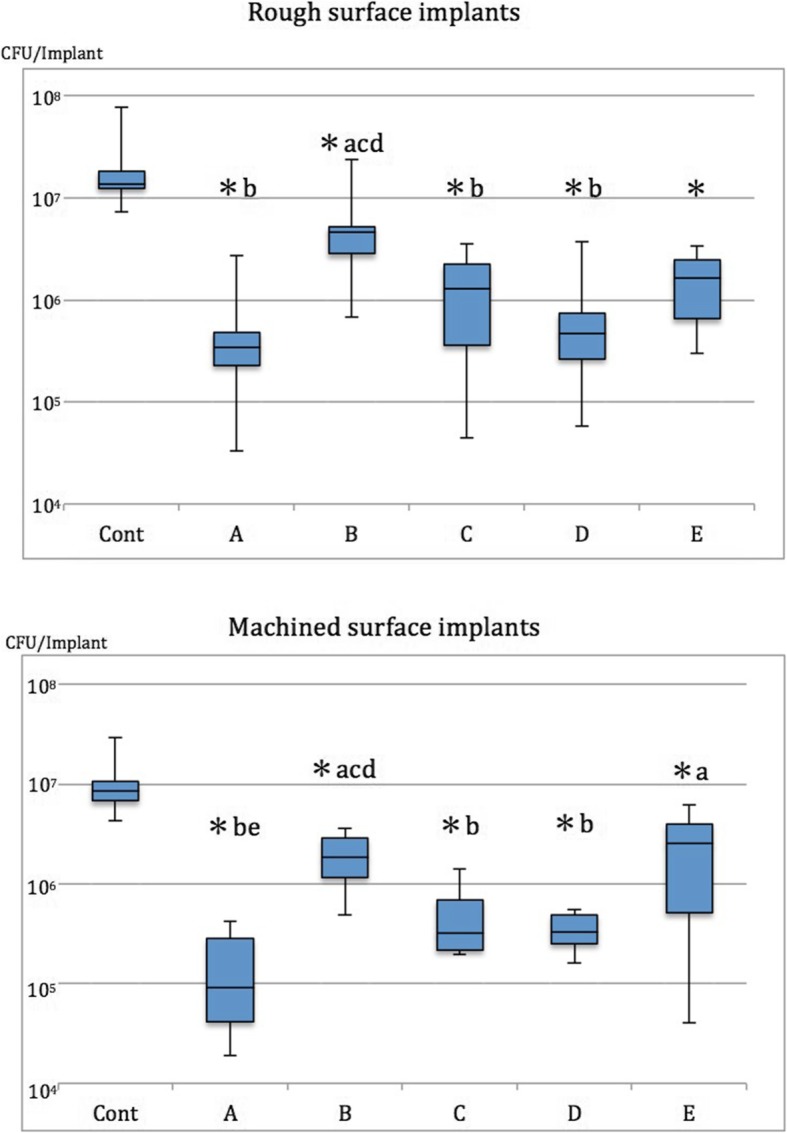
Quantitative analysis of CFU counts on rough and machined surface implants after cleansing by each method. Asterisk represents vs Cont; **a**, vs G; **b**, vs US; **c**, vs Air; **d**, vs Rot; **e**, vs Las which indicates *p* < 0.05

**Fig. 6:**
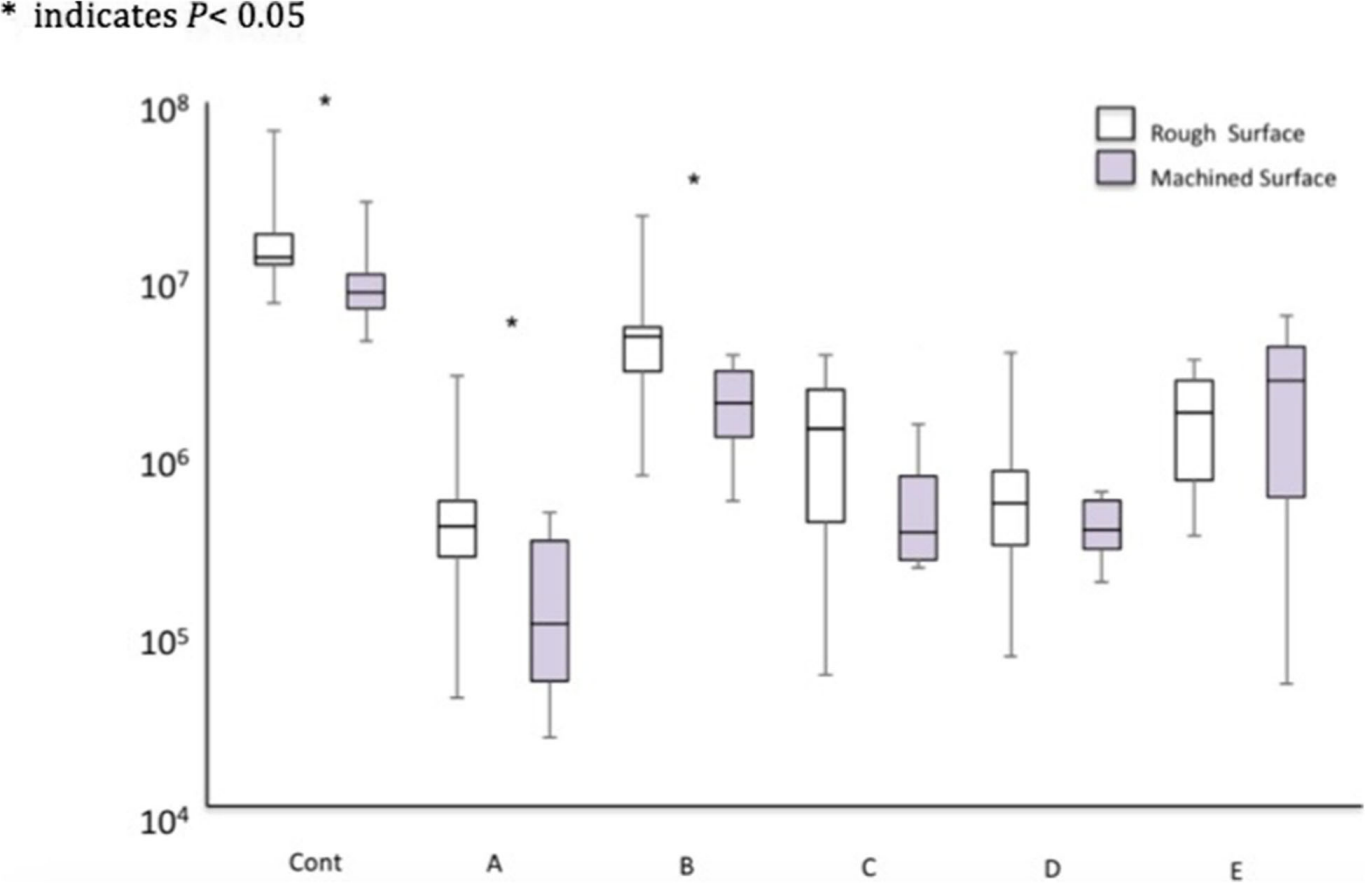
Comparison of cleansability of each decontamination method on the different implant surfaces. Asterisk indicates *p* < 0.05

**Table 3:** Quantitative analysis of CFU counts (× 10^5^) from rough and machined surface implants after cleansing by each method

**Rough surface**	**Cont**	**G**	**US**	**Air**	**Rot**	**Las**
**Median**	137.5	3.4	46.5	13.0	4.8	16.3
**Min**	73.0	0.3	6.8	0.5	0.6	3.0
**Max**	785.0	27.0	240.0	35.5	37.0	34.0
**Machine surface**	**Cont**	**G**	**US**	**Air**	**Rot**	**Las**
**Median**	84.5	0.9	8.5	3.2	3.3	25.3
**Min**	43.0	0.2	0.9	2.0	1.6	0.4
**Max**	295.0	4.2	36.0	14.0	5.6	61.5

#### Machined surface implants (Fig. 5, Table 3)

All decontamination methods showed significantly better cleansability in terms of bacterial CFU counts compared with Cont (*p* < 0.05). Additionally, G, Air, and Rot demonstrated significantly better cleansability than US (*p* < 0.05), and only G showed significantly better cleansability compared with Las (*p* < 0.05).

#### Rough vs. machined surface implants (Fig. 6, Table 3)

Cont, G, and US demonstrated significant differences in bacterial CFU counts between rough and machined surface implants (*p* < 0.05). Although there was no significant difference in CFU counts between the two groups following Air, Rot, and Las application, machined surface implants appeared to show lower CFU counts than rough surface implants following Air and Rot application.

## Discussion

### Study design

This study was performed following an ex vivo design to overcome the drawbacks of previous studies. One particular difference in this study was the use of a commercially available screw-shaped implant. As the implant shape and design have rather complicated macro- and microstructures compared with titanium disks or different forms of titanium commonly used in experimental studies, previous results could not be easily interpreted and extrapolated to the clinical setting [14, 19]. However, the use of genuine implants allowed us to evaluate the cleansability of each method on contaminated implant surfaces. Without any limitations of accessibility and visibility, the efficacy of each decontamination method could be evaluated in a limited time frame.

Another critical difference in this study was the evaluation of dental plaque on implant surfaces collected in the mouth of participants rather than a single bacterial species [14, 20] or artificial biofilms [21, 22]. Dental plaque comprises 700–1000 bacterial species and is significantly different to a single bacterial colony or artificial biofilms. By assessing CFU counts via culture technique, the ability of each method to physically disrupt oral biofilms on contaminated implant surfaces could be evaluated. Conversely, a limitation of this study is that biofilms that cause peri-implantitis differ from those evaluated in this study. The biofilms in peri-implantitis form in anaerobic deep submucosal areas [23]. However, in this study, only supragingival oral biofilms could accumulate on the mounted implant surfaces. It seems difficult to reproduce the same quality of submucosal biofilms around implants, which are thought to be an etiological factor of peri-implant disease in the laboratory [24]. To date, there are a few studies that have tried to reproduce submucosal biofilms [22, 25], but such systems have not yet been completely established. This limitation of the present study should be kept in mind.

To the best of our knowledge, there is only one study that has used a similar experimental design to that of this study. Augthun et al. [10] examined the cultivability of mouse fibroblasts after cleansing machined or plasma-splayed surface implants carried on acrylic plates that had been contaminated with supragingival plaque from individuals. A plastic hand scaler and an air-abrasive system with sodium bicarbonate powder were employed in their study. A similar number of viable fibroblasts were observed after cleansing the implant with the air abrasive as the non-contaminated control implant. However, the number of viable cells was significantly reduced on the implant cleansed with the plastic scaler. This study had two drawbacks. First, they did not employ a quantitative analysis to evaluate the cleansing effect. Second, the SEM analysis used to evaluate the cleansing effect was too low (10- to 100-fold). In the present study, the presence of residual biofilms after instrumentation was determined using higher magnification SEM analysis (up to 5000-fold) and CFU counts. In this context, our findings may provide more accurate evidence than that demonstrated by the aforementioned study.

### SEM analysis

Based on the results of the SEM qualitative analysis, gauze soaked in saline and the rotary stainless steel instrument consistently showed good cleansability on rough and machined surface implants compared with the other methods. Conversely, the Er:YAG laser showed inferior cleansability to all other methods especially on rough surface implants. The ultrasonic scaler and air abrasive exhibited fair to good cleansability on both surface implants. Generally, the cleansability of each method appeared to be better on machined surface implants than rough surface implants.

The cleansability of gauze soaked in saline has previously been evaluated with and without antiseptics in vitro and in vivo [6, 9, 23, 26]. Charalampakis et al. [23] examined the efficacy of mechanical and chemical decontamination methods using titanium disks contaminated intraorally for 4 days. They employed four decontamination methods: gauze in saline, chlorhexidine, delmopinol, and an essential oil mixture. The SEM analysis demonstrated that three different rough surface disks harbored complex and firmly attached biofilms after gauze scrubbing irrespective of which antiseptic or saline was used. However, the disks with a turned surface hosted fewer biofilm clusters after scrubbing. This finding is in line with our result showing the better cleansability of gauze soaked in saline on the machined surface implants compared with the rough surface implants. The ultrasonic scaler, air abrasives, and Er:YAG laser have also been well investigated and used for the treatment of peri-implantitis [7, 27]. Schmage et al. [28] revealed the high cleansability of air abrasives and considerable cleansability of ultrasonic scalers and Er:YAG laser on titanium disks contaminated by a biofilm layer of *Streptococcus mutans*. The cleaning score of the air abrasives was the highest, and two types of ultrasonic scaler with a non-metal tip and the Er:YAG laser showed medium cleaning scores but better cleansability than non-metal curettes or a prophylaxis brush/cup. In the present study, the ultrasonic scaler displayed modest results. The tip used in this study was specially designed for cleansing contaminated implants with complicated macro- and microstructures. As the tip dimension was small in order to cleanse very narrow spaces, such as the valley of micro- or macrothreads, good cleansability was expected to be seen in such areas. This method could remove biofilms from small areas, but not in their entirety and not from the valley of microthreads. Moreover, the overall effect of biofilm removal did not appear impressive. One possible explanation for this result is that a treatment time of 1 min was not sufficient to use this small tip effectively. If more time was given to the ultrasonic scaler group, it might be possible to eliminate more biofilms, especially from microstructured areas of the implant.

Regarding the air abrasives, the cleansing effect in the SEM analysis was also as considerable as that achieved by the ultrasonic scaler in the present study, in contrast to the results of the aforementioned study. Louropoulou et al. [29] also stated in their systematic review that an air-powder abrasive system with sodium bicarbonate powder could cleanse contaminated rough/smooth implant surfaces without losing biocompatibility compared with a plastic scaler, metal curette, rotating titanium brush, and ultrasonic scaler. In the present study, the air abrasive showed fair to good cleansability with glycine powder but did not achieve the best result among the tested decontamination methods. The reason for this difference may be associated with the different experimental conditions (e.g., cleaning time, powder, power setting, and nozzles). Although free access to the genuine implant surface in the present study allowed us to evaluate the efficacy of each decontamination method, glycine powder as an air abrasive may not have the best cleansing potential among the tested methods. In the present study, the Er:YAG laser generally showed inferior cleansability. Er:YAG lasers have also been used in non-surgical [27] and surgical [30] peri-implantitis treatment. It was previously reported that implant surface decontamination by Er:YAG lasers demonstrated good cleansability of the contaminated implant surface compared with other decontamination methods [31–33]. The reason for the inferior cleansability of the Er:YAG laser observed in this study compared with the other decontamination methods is discussed below; however, dense biofilms remained on rough surface implants in particular after decontamination by the Er:YAG laser.

A rotary stainless steel instrument has a small head composed of stainless steel that allows clinicians good accessibility to deep intrabony defect areas. To the best of our knowledge, no study has clarified the cleansability of this rotary stainless steel instrument. In the present study, it was shown that it might be useful for cleansing contaminated implant surfaces. However, the rotary stainless steel instrument created numerous shallow scratches, especially on machined surface implants. John et al. [12] compared the supragingival plaque cleansability of a rotary titanium instrument to that of a stainless metal curette on contaminated titanium disks. The residual biofilm area left on implant treated with the rotary titanium instrument was significantly lower than in the stainless metal curette, and the surface alteration of the titanium disks could not be shown in SEM analysis. Although the cleansability of the rotary stainless steel instrument in the present study is superior and advantageous, the downside of the surface alteration is an issue to consider.

It has been previously stated that the alteration of the implant surface during cleansing may attenuate biocompatibility [29]. However, several clinical studies revealed the considerable treatment effect even though there was certain expected damage on the implant surface [7, 34]. Therefore, it is assumed that the most important consideration for treating peri-implantitis in the clinical setting should be to improve the cleansability of any instrumentation to effectively remove biofilms irrespective of implant surface alteration.

### Analysis of bacterial CFU count

In the present study, the gauze soaked in saline, rotary stainless steel instrument, and air abrasive demonstrated significantly greater cleansability to remove biofilms compared with the ultrasonic scaler on rough and machined surface implants. Generally, gauze soaked in saline appeared to possess the best cleansability among all the tested decontamination methods although there was no significant difference among the three methods with the greatest cleansability (G, Rot, Air). In the analysis between the two surfaces, surface characteristics significantly influenced total CFU counts between rough and machined surface implants when testing the control and gauze soaked in saline and ultrasonic scaler. Overall, machined surface implants tended to show lower CFU counts than rough surface implants apart from those treated with the Er:YAG laser.

Charalampakis et al. [23] examined the effectiveness of mechanical and chemical decontamination methods using titanium disks contaminated intraorally. They employed four decontamination methods: gauze in saline, chlorhexidine, delmopinol, and an essential oil mixture. The authors discovered there was no significant difference in CFU counts among the four methods. In the present study, our findings were in line with their report regarding the difficulty of removing biofilms from contaminated titanium surfaces. Even mechanical decontamination with a chemical agent did not yield any significant difference in CFU counts in their study. It has also been revealed that chemical agents in conjunction with mechanical debridement on contaminated implants could not augment a significant treatment effect [24]. This is one of the reasons why we focused on mechanical decontamination methods to cleanse the contaminated implant surfaces.

Sahrmann et al. [15] tested three instruments (ultrasonic scaler, Gracey curette, and air abrasive device with glycine powder) on rough surface implants stained with indelible ink used as artificial plaque. There was a statistically significant difference in terms of stain removal rate. The air abrasive device showed the best result among the tested instruments. The result of this study is in line with our result showing the superiority of the air abrasive compared with the ultrasonic scaler.

Widodo et al. [14] evaluated the efficacy of different methods used to cleanse titanium disks contaminated by *S. aureus* biofilm in vitro. They used the following methods: (i) rinsing with phosphate-buffered saline, (ii) rinsing with chlorhexidine digluconate 0.2%, (iii) application of photodynamic therapy (iv), use of a cotton pellet, (v) use of a titanium brush, and (vi) the combination of a titanium brush and photodynamic therapy. The results showed that the use of a titanium brush with/without photodynamic therapy was more effective in reducing the bacterial load on both polished and rough titanium implant surfaces than the other methods. Our results are also in accordance with their results in terms of the high cleansability of the rotary metal instrument. In addition, the cotton pellet showed moderate cleansability among the tested methods, but the cleansing time for the cotton pellet (60 s) was shorter than that of the titanium brush with (120 s + 60 s)/without (120 s) photodynamic therapy. If adjusting the difference of cleansing time, the cotton pellet might show equivalent cleansability to the titanium brush.

In contrast to the past in vivo and in vitro studies [35, 36], the Er:YAG laser demonstrated an inferior cleansability on the contaminated implant surfaces. The Er:YAG setting (60 mJ/pulse, 10 pps) in the present study was within the normal recommended range for cleansing an implant surface without causing damage to the implant surface or the peri-implant tissue cells [37–39] and to ensure the safety of peri-implant tissue [37]. Kreisler et al. [11, 37] used the same setting to cleanse a contaminated implant surface but without water coolant and demonstrated a good result. The reason why we could not achieve the same result might be associated with the water coolant used for further safety reasons in our study. In the clinical setting, the Er:YAG laser has been applied to treat peri-implantitis [27, 30, 40]. However, one report cautioned that the use of Er:YAG laser treatment as a non-surgical therapy had previously led to trauma of the peri-implant soft tissue, thereby causing unnecessary recession of the peri-implant mucosa [30]. In this context, when the Er:YAG laser is applied to the treatment of peri-implant disease, water coolant should be considered for safety. There are many aspects that contribute to the efficacy of the Er:YAG laser (e.g., setting, coolant, tip distance from the tip to the contaminated implant surface). Such differences should be investigated in future studies.

### Surface characteristics

Through SEM analysis and CFU counts, it was demonstrated that, except for the Er:YAG laser, decontamination of the machined surface implant was easier than that of the rough surface implant regardless of decontamination method. Gauze soaked in saline and the ultrasonic scaler demonstrated a statistically significant difference in CFU counts between the two surfaces. In this context, a machined surface implant may be advantageous for recovering biocompatibility after cleansing the contaminated implant surface. In a randomized controlled trial, Carcuac et al. [6] demonstrated greater treatment success in a machined surface implant group than a modified surface implant group. The present study may support this clinical result, and the application of gauze soaked in saline may be regarded as a gold standard technique to cleanse a machined surface implant.

## Conclusions

In the present ex vivo experimental study, none of the tested decontamination methods thoroughly eliminated biofilms formed on rough/machined surface implants intraorally. Gauze soaked in saline and the rotary stainless steel instrument showed better cleansability than the ultrasonic scaler in qualitative and quantitative analyses and may be advantageous for cleansing contaminated implant surfaces. Additionally, except for the Er:YAG laser, each of the tested decontamination methods appeared to be more effective on machined surface implants than rough surface implants in terms of reducing biofilms qualitatively and quantitatively. Research on the optimum combination of different cleansing methods that compensate for each method’s respective downsides is urgently required. Further research is needed to elucidate the most effective method to cleanse contaminated implant surfaces.

## Data Availability

The datasets used during the current study are available from the corresponding author on reasonable request.

## Abbreviations

Cont: Control (no decontamination)
G: Gauze soaked in saline
US: Ultrasonic scaler (SUPRASSON P-MAX, Satelec-Acteon Group, Bordeaux, France; power setting: P5, tip: Implant Protect IP3L/R)
Air: Air abrasive (AIR-FLOW MASTER PIEZON®, EMS, Nyon, Switzerland; power setting: water flow 100%, air pressure 75%, powder: AIR-FLOW® PERIO POWDER, nozzle: PERIO-FLOW® nozzles, distance from the nozzle to the implant 2 mm)
Rot: Rotary stainless steel instrument (iBrush, NeoBiotech©, Los Angeles, CA, USA; rotating speed 1500 rpm)
Las: Er:YAG laser (Erwin AdvErL, J.Morita©, Kyoto, Japan; power setting 60 mJ/pulse, 10 pps, tip: C600F, distance from the tip to the implant 2 mm)
